# Exploring the interplay between mitochondria and endoplasmic reticulum in pulmonary arterial hypertension

**DOI:** 10.3389/fcvm.2025.1623775

**Published:** 2025-11-05

**Authors:** Zhipeng Liao, Yuanzhou He

**Affiliations:** ^1^ Department of Respiratory and Critical Care Medicine, Department of Internal Medicine, Tongji Hospital, Tongji Medical College, Huazhong University of Science and Technology, Wuhan, China; ^2^ The Second Clinical Department, Tongji Medical College, Huazhong University of Science and Technology, Wuhan, China

**Keywords:** pulmonary arterial hypertension, mitochondria-associated endoplasmic reticulum membranes, mitochondrial dynamics, calcium, ER stress

## Abstract

Pulmonary arterial hypertension (PAH) is a subtype of pulmonary hypertension (PH), characterized by pulmonary arterial remodeling. This disease frequently progresses to right heart failure and can result in patient mortality. Research at the cellular and molecular level is gradually revealing the mechanism underlying the development of pulmonary arterial hypertension, providing new avenues for treatment by identifying potential therapeutic targets. Contact between the endoplasmic reticulum and mitochondria has been recognized for several decades. And an increasing number of laboratory and clinical studies are beginning to elucidate the relationship between PAH and the interplay involving mitochondria and the endoplasmic reticulum. In this review, we first introduce the basic normal biological functions and processes of MAM-based mitochondrial-endoplasmic reticulum interactions. We then discuss how the dysfunction contributes to pulmonary arterial hypertension (PAH), focusing on three key aspects, mitochondrial dynamics, calcium homeostasis, and endoplasmic reticulum stress. Clarifying these issues may provide important insights for therapeutic interventions in PAH.

## 1 Introduction

Pulmonary arterial hypertension is a life-threatening disorder characterized by elevated pressure in the pulmonary arteries due to increased pulmonary vascular resistance (1). PAH is a clinical subtype of PH (pulmonary hypertension), and the remaining four types include PH due to left heart disease, PH due to chronic lung disease, chronic thromboembolic pulmonary hypertension, and PH with unclear mechanisms and/or multifactorial causes (2). Currently, the international definition for pulmonary hypertension (PH) is an average pulmonary artery pressure exceeding 20 mmHg during right heart catheterization while the patient is at rest (3). Although PAH is considered a relatively rare disease, its incidence and prevalence have been increasing in recent years (4). According to a large epidemiological analysis of pulmonary arterial hypertension based on the Global Burden of Disease Study, from 1990–2021, the total number of DALYs (disability-adjusted life years) caused by pulmonary arterial hypertension worldwide decreased by 6.6%. Despite an overall reduction in burden, PAH-related DALYs increased by 13.9% in high SDI (socio-demographic index) countries. Meanwhile, global deaths due to PAH rose by 48.5% during this period (5). Although the pathophysiology of pulmonary arterial hypertension (PAH) remains incompletely understood, scientists have made progress in several areas, such as metabolic reprogramming (6), inflammatory effects (7), organelle communication (8), microRNAs (9), and ferroptosis (10). Given the crucial roles these targets play in the development of PAH, new drug strategies and delivery methods are continuously being developed and are showing improvements in both animal models and human trials (11).

Mitochondria are continually being explored for their functions and behaviors, and their roles in diseases such as cancer, diabetes, and vascular disorders have been extensively studied (12–14). The endoplasmic reticulum (ER) is a cellular organelle responsible for protein synthesis, folding, and transport, as well as lipid metabolism and calcium storage (15). In recent decades, research into interorganelle communication has become increasingly sophisticated, even evolving into a new field of study known as Contactology (16). In this theory, mitochondria and the endoplasmic reticulum (ER) have long been considered functional and structural units (8, 16, 17). Together, these two organelles are involved in a variety of biological functions, such as the regulation of mitochondrial dynamics, metabolic regulation and maintenance of calcium homeostasis, as well as biological responses such as ER stress and inflammation. MAMs (Mitochondria-associated endoplasmic reticulum membranes), initially viewed as key membrane structures for lipid synthesis and transport between the endoplasmic reticulum and mitochondria, are increasingly recognized as linking these two organelles in multiple biological functions, thereby maintaining cellular homeostasis (18, 19). The role of MAMs in neurological disorders, endocrine disorders, and cancer has been extensively studied, and drugs targeting them are relatively well established (13, 20, 21). This review aims to provide an overview of the normal biological functions of mitochondria and endoplasmic reticulum interaction, as well as their roles in pulmonary arterial hypertension.

## 2 ER-mitochondria interactions in normal cellular functions

Mitochondrial-endoplasmic reticulum coupling was originally discovered in a teleost (22). Furthermore, in the 1950s, scientists used electron microscopy to observe two organelles spatially connected in rat liver cells (23). At that time, MAMs were initially thought to be key sites of lipid synthesis, a specific membrane structure and protein enrichment site that scientists called fraction X (24). Since then, Innovations and applications in biochemical techniques and research methodologies have enhanced our understanding of the structure and function of MAMs. These advancements have provided deeper insights into the critical roles that MAMs play in regulating cellular homeostasis and their involvement in various pathological conditions (25, 26). More than 1,000 distinct proteins may localize to the ER-MAMs, forming complexes that regulate the structure and function of these subcompartments (27). In the following section, we will discuss several key biological processes involving mitochondria-endoplasmic reticulum interactions, with a focus on MAMs, in a point-by-point manner. And we summarize here a table to show the biological roles of MAMs and their key proteins, as well as to complement the sections not detailed in the main text (Table 1).

**Table 1:** Biological role of MAMs and their key proteins.

Biological function	Key proteins	References
Lipid Metabolism and Transportation	PSS1/2, ORP5/8, Mfn2, CDS2, VAPB-PTPIP51, ACAT1, Caveolin 1	(134–136)
Ca^2+^ transfer	IP3R, RyR, GRP75, VDAC, MCU, Mfn2, Sig-1R, VAPB, PTPIP51	(48)
Mitochondrial dynamics	Mfn1/2, Drp1, Opa1, FUNDC1	(137, 138)
ER stress	Mfn2, IRE1, PERK, ATF6	(139)
Inflammation	VDAC, PACS2, FUNDC1,	(140, 141)
Autophagy	ATG14, STX17, ERLIN1-AMBRA1	(139, 142, 143)
Apoptosis	Mfn2, Fis1, PACS2, Bcl-xL	(43, 144, 145)

### 2.1 Mitochondrial dynamics

Having an evolutionary relationship with an ancient bacterium, mitochondria are semiautonomous organelles (28). Mitochondrial dynamics refer to the processes of fission, fusion, mitophagy, and transport, which are crucial for optimal signaling and metabolic functions (29). These dynamic processes are believed to be closely related to the membrane contact sites with the endoplasmic reticulum, and scientists believe that the contact site is the regulatory and participatory node for the bidirectional dynamics of mitochondrial fission and fusion (30).

Mitochondrial fission is essential for regulating mitochondrial morphology, distribution, and quality control, enabling the segregation and removal of damaged mitochondria through mitophagy, and plays critical roles in apoptosis, cellular metabolism, and development (31). The initiation of mitochondrial fission occurs in MAMs. Using tomography and fluorescence microscopy, Friedman *et al*. directly observed the specific structures of these contacts and concluded that the ER marks the division site and maintains contact with the mitochondria throughout the entire fission process (32). Mitochondrial fission mainly involves three steps: (a) marking the fission site, (b) Dynamin-related protein 1 (Drp1) assembling into a helical superstructure around the marked fission site, and (c) GTP hydrolysis subsequently causing Drp1 helical contraction, thereby triggering mitochondrial fission (33). The ER-bound protein INF2 and the mitochondrial actin-nucleator Spire1C form a complex that promotes Myosin IIa assembly to generate the mechanical force for pre-constriction, followed by recruitment of Drp1—*via* receptors MFF, MiD49, and MiD51—to the constricted site for further membrane constriction and division, with dynamin 2 (DYN2) potentially contributing to the final scission step, though its essential role remains debated (34, 35).

**Figure 1:**
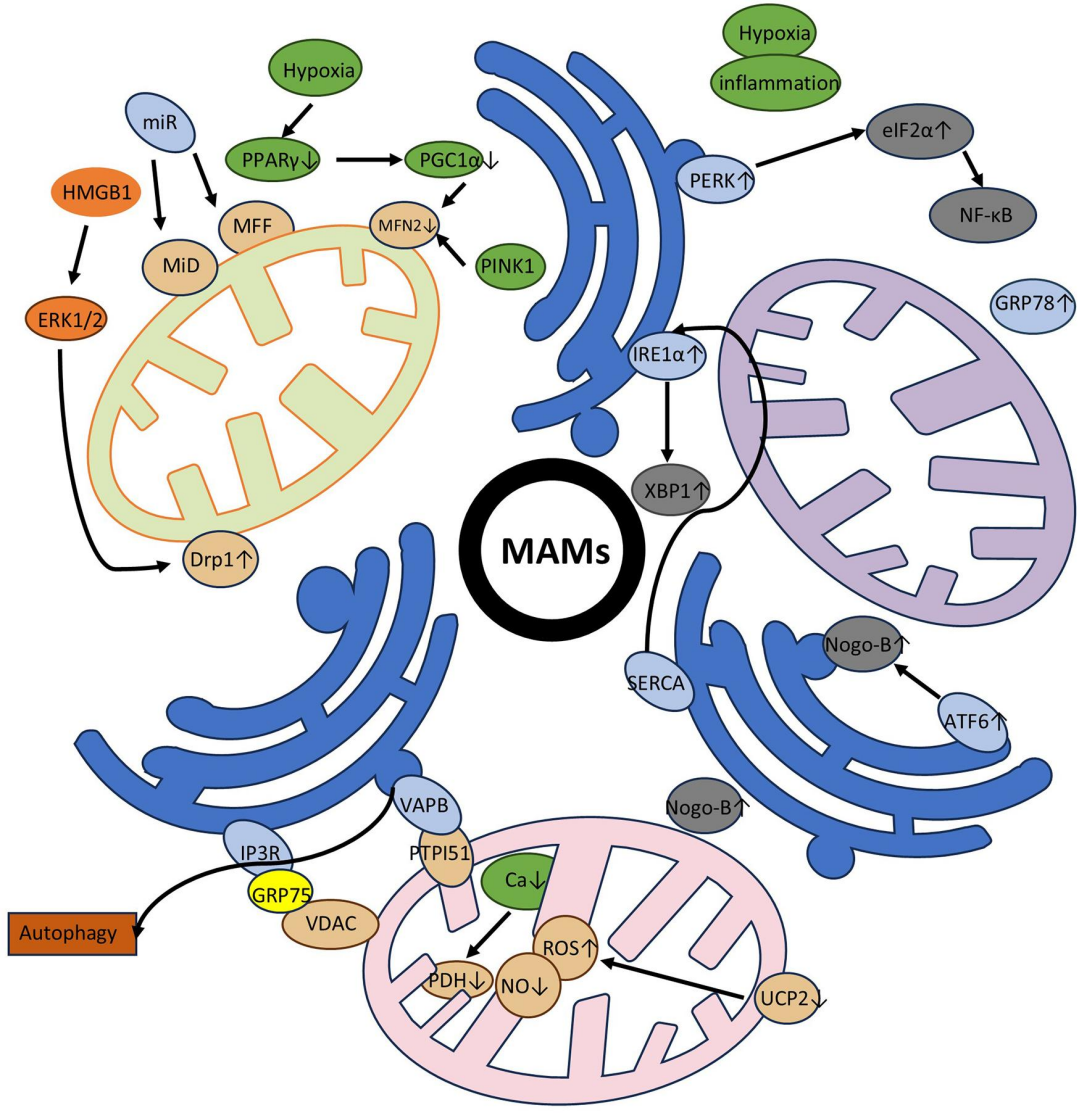
Specific processes and involved proteins in mitochondrial fission and fusion. The schematic illustrates the specific proteins and processes involved in mitochondrial division and fusion, and is used to supplement the section in the main text on mitochondrial dynamics overview. The relevant proteins and organelles involved are displayed in the upper right corner of the image. It is well known that the accumulation of Drp1 at mitochondrial endoplasmic reticulum contact sites is a central mechanism for mitochondrial contraction and division. Previously, mitochondrial precontraction was not possible without the ER-bound protein inverted form 2 (INF2) and the actin-nucleating mitochondrial anchor Spire1C. Spire1C and INF2 form a complex that enhances the assembly of Myosin IIa in the contact site, which provides the critical mechanical contractile force. The mitochondrial Drp1 receptors are MFF, MiD49, and MiD51. After Drp1 is recruited and assembled, further contraction of the membrane is carried out. Some scientists have also suggested that the endocytic-related dynamin 2 (DYN2) protein is also involved in this final step of division, although the necessity of a related mechanism remains questionable. During mitochondrial fusion, it is clear that the outer membrane is preferred over the inner membrane and different proteins are involved. The fusion of the outer membrane is involved by Mfn1, Mfn2, while the fusion of the inner membrane is mediated by OPA1. We have made clear the importance of the ER in this, in particular by demonstrating that Mfns are localized to membrane contacts and that ER tubules mark sites of mitochondrial fusion. To represent this bidirectional mitochondrial dynamic process, we use black arrows to form a loop to indicate this.

Mitochondrial fusion forms a continuous mitochondrial network to maintain mitochondrial functional homeostasis, promote metabolic coordination, and DNA complementation (36). Similarly, researchers have discovered that mitochondrial fusion is closely associated with contact sites on the endoplasmic reticulum, as evidenced by the co-localization of related proteins and ER tubules at mitochondrial fusion sites (37). The mitochondrial fusion process involves fusion of the outer membrane and fusion of the inner membrane. The fusion of the outer mitochondrial membrane (OMM) and inner mitochondrial membrane (IMM) is driven by integral membrane proteins that form a dimeric antiparallel structure, with OMM fusion primarily mediated by Mfn1(Mitofusin 1) and Mfn2, and IMM fusion mainly dependent on OPA1 (14). To reflect the rigor and completeness of the review, specific proteins and detailed processes regarding mitochondrial fission and fusion will be given in the supplementary figure and their accompanying explanations (Figure 1).

### 2.2 Calcium homeostasis and calcium communication

Calcium (Ca^2+^) functions as a critical second messenger involved in the regulation of diverse intracellular processes. Intracellular Ca²^+^ levels influence a wide range of biological functions, including metabolic regulation, gene transcription, cell proliferation, migration, and apoptosis (38, 39). ER acts as the main intracellular Ca²^+^ reservoir and is structurally connected to the outer mitochondrial membrane (OMM) via MAMs (40). The efficiency of Ca²^+^ transfer between the ER and mitochondria is influenced by the physical distance between the two organelles. Studies have shown that when this distance increases to approximately 15 nm, Ca²^+^ transfer becomes more efficient (41, 42). For specific proteins, the IP3Rs-GRP75-VDACs complex is considered a critical component involved in calcium ion transfer from the endoplasmic reticulum to the mitochondria in MAMs (13, 43). IP3Rs serve as channels for calcium efflux from the endoplasmic reticulum (44, 45). VDACs are localized in the outer mitochondrial membrane and mediate the exchange of substances across the mitochondria, including calcium ions (46, 47). IP3R and VDAC physically and functionally interact through GRP75 (48). Additionally, the GRP78 protein in MAMs forms a complex with sig-1R on the ER, which under certain conditions dissociates to increase Ca²^+^ transfer via the IP3R pathway (49, 50). In addition to IP₃Rs, ryanodine receptors (RYRs)—another class of Ca²^+^ release channels—are also present at MAMs and contribute significantly to inter-organelle Ca²^+^ signaling (51, 52). Notably, IP3R-mediated Ca²^+^ signaling has also been linked to other MAM-resident proteins, such as vesicle-associated membrane protein-associated protein B (VAPB) and PTPIP51, both of which are involved in maintaining ER–mitochondria tethering and regulating Ca²^+^ flux under various cellular conditions (53). Key proteins involved in Ca²^+^ handling also include sarco/endoplasmic reticulum Ca²^+^-ATPase (SERCA) pumps, which actively transport Ca²^+^ from the cytoplasm into the ER lumen to maintain ER Ca²^+^ homeostasis (54). Upon Ca²^+^ release from the ER, a rapid increase in cytoplasmic Ca²^+^ occurs, triggering immediate buffering by both cytoplasmic and organelle Ca²^+^ uptake systems (55).

The mitochondrial calcium uniporter (MCU) is a highly selective calcium channel embedded in the inner mitochondrial membrane (IMM) that constitutes the primary pathway for calcium entry into mitochondria. The MCU complex forms a multimeric protein complex with regulatory subunits that fine-tune its activity according to cellular energy demands and calcium signaling requirements (56). The core MCU protein forms the conductive pore, while essential regulatory subunits include Mitochondrial Calcium Uptake 1 and 2 (MICU1, MICU2) and the Essential MCU Regulator (EMRE). Also, Mitochondrial calcium efflux is equally crucial for maintaining appropriate matrix calcium levels and is primarily mediated by sodium-calcium exchange mechanisms (57). For decades, the molecular identity of the mitochondrial Na^+^/Ca²^+^ exchanger (mito-NCX) remained controversial, with NCLX proposed as a candidate but failing to fully explain observed physiological behaviors due to its lack of Na^+^ binding sites and inconsistent knockout phenotypes (58).

Calcium signaling within mitochondria directly regulates key metabolic enzymes that control flux through the tricarboxylic acid (TCA) cycle and electron transport chain. Three dehydrogenases show particular sensitivity to calcium-mediated activation. Pyruvate dehydrogenase phosphatase (PDP) activates the pyruvate dehydrogenase complex (PDC) through dephosphorylation, allowing increased conversion of pyruvate to acetyl-CoA (59). Calcium binding to PDP enhances its activity, thereby promoting glycolysis-derived entry into the TCA cycle during increased energy demand. Isocitrate dehydrogenase (NAD+-ICDH) and α-ketoglutarate dehydrogenase (OGDH) are both activated by increased mitochondrial calcium, enhancing reducing equivalent (NADH and FADH₂) production and thus stimulating electron transport and ATP synthesis (60). The coordinated activation of these enzymes by calcium ensures that energy production matches cellular activation states. The FAD-dependent glycerol phosphate dehydrogenase (FAD-GPDH) shuttle, which transfers reducing equivalents from cytosol to mitochondria, is also calcium-sensitive, allowing integrated regulation of cytosolic and mitochondrial metabolic processes. The F₁–F_o_ ATP synthase complex, which catalyzes the final step of oxidative phosphorylation by producing ATP from ADP and inorganic phosphate, is similarly regulated by calcium signaling (61). Calcium indirectly modulates ATP synthase activity through effects on the electrochemical gradient and substrate availability, though recent evidence suggests more direct regulatory mechanisms may exist.

Mitochondrial metabolism is closely intertwined with the regulation of calcium ions. Calcium homeostasis in both the mitochondria and cytoplasm plays a crucial role in modulating enzyme activity, including those involved in glucose metabolism (62). Another example is that VAPB and PTPIP51, mentioned earlier, play a key role in calcium signaling and affect energy production in mitochondria (63).

### 2.3 ER stress

The endoplasmic reticulum (ER) is a central organelle responsible for protein folding, lipid synthesis, and calcium homeostasis in eukaryotic cells. ER stress refers to the accumulation of unfolded or misfolded proteins within the ER, which occurs when the cellular demand for protein processing exceeds the capacity of the ER quality control machinery. This pathological condition can be triggered by various physiological and pathological insults, including nutrient deprivation, metabolic disturbances, oxidative stress, DNA damage, and certain infections. If unresolved, ER stress leads to cellular dysfunction through mechanisms such as inflammation, apoptosis, and mitochondrial impairment (62–64). Three endoplasmic reticulum transmembrane proteins act as sensors of endoplasmic reticulum stress: activating transcription factor 6 (ATF6), inositol-requiring enzyme 1 alpha (IRE1 α) and PRKR-like endoplasmic reticulum kinase (PERK). Under normal conditions, the molecular chaperone BiP (Binding Immunoglobulin Protein; also known as GRP78) binds to ER stress sensors (e.g., IRE1α, PERK, and ATF6), maintaining their inactive state. During endoplasmic reticulum (ER) stress, BiP dissociates from these sensors due to the accumulation of misfolded proteins in the ER (65). PERK is uniquely enriched in MAMs and is also closely associated with ROS-mediated stress (66). Similarly, the mitochondrial ubiquitin ligase (MITOL) inhibits ER stress-induced apoptosis by ubiquitinating IRE1α at MAMs (67). Traditionally considered closely related to MAMs, Nogo B can be activated by the ATF6 pathway, leading to increased expression and disruption of MAMs (68).

### 2.4 Lipid synthesis and transfer

Phosphatidylserine (PS), an essential anionic phospholipid for the structural and functional integrity of cell membranes, is synthesized by two distinct enzymes, phosphatidylserine synthases-1 (PSS1) and -2 (PSS2), which are located in MAMs (69, 70). In addition to its synthesis, PS transport at the MAM interface involves oxysterol-binding protein (OSBP)-related proteins ORP5 and ORP8. These proteins are thought to mediate PS transfer between membranes and interact with PTPIP51, a mitochondrial outer membrane protein that contributes to ER-mitochondria tethering (71). Another key player in lipid metabolism within MAMs is acetyl-CoA cholesterol acyltransferase 1 (ACAT1), which plays a central role in cholesterol esterification and homeostasis. ACAT1 is highly enriched in MAMs, where it facilitates cholesterol storage and trafficking (72, 73). Furthermore, caveolin 1 is also enriched in MAMs. Caveolin 1 interacts closely with ACAT1, inserts into the endoplasmic reticulum membrane, and participates in cholesterol transport. In addition, it contributes to the formation of cholesterol-rich signaling platforms, thereby influencing lipid signaling and membrane organization (27, 74).

### 2.5 Inflammation

The link between MAMs and inflammation lies in the activation of the NOD-like receptor protein 3 (NLRP3) inflammasome (75). Calcium signaling plays a crucial role in the activation of the NLRP3 inflammasome (76). Studies have shown that mitochondrial dynamics also contribute to this inflammatory response. For instance, Misawa et al. found that microtubule-driven mitochondrial migration is relevant (77). Additionally, the Mfn2 protein has been associated with NLRP3 inflammasome activation following viral infection (78). In addition to Mfn2, VDAC has also been proposed to participate in NLRP3 inflammasome assembly. VDAC may facilitate the cross-talk between ER-derived Ca²^+^ signals and mitochondrial stress responses, thereby contributing to inflammasome formation and downstream pro-inflammatory signaling (79).

## 3 Dysregulated mitochondrial-ER interplay in PAH

### 3.1 Inappropriate mitochondrial dynamics

Many studies have focused on the role of imbalanced mitochondrial dynamics in PAH (14, 80). In this section, we describe two key proteins involved in division and fusion-Drp1 and Mfn2. A significant portion of current research centers on Drp1. Excessive Drp1-mediated mitochondrial fission has been found in cells associated with pulmonary arterial hypertension, including pulmonary artery smooth muscle cells (PASMCs) (81) and pulmonary artery adventitial fibroblasts (82). Similar therapeutic effects have been reported in animal models. In rats co-administered with Mdivi-1 and CoCl_2_, not only was there a recovery of exercise capacity, but there was also a significant improvement in PAAT (pulmonary arterial acceleration time) (83). Drp1 requires association with adapter proteins to trigger initiate fission (84). One key mechanism involved in the pathogenesis of PAH is that decreased expression of miR-34a-3p leads to upregulation of MiD, which in turn increases mitosis in PASMC, driving pathological proliferation and resistance to cell apoptosis, and simultaneously, the effectiveness of *in vivo* nebulization of MiDs and miR-34a-3p was demonstrated, showing their ability to attenuate experimental PAH and reduce cell proliferation (85). In addition to MiD, Huang *et al*. reported that miR-340-5p regulates the MFF-SIRT1/3 axis to improve mitochondrial homeostasis and increase the imbalance between proliferation and apoptosis in hypoxia-treated PAMSCs, providing a theoretical basis for the prevention and treatment of PAH (86). Drp1, when phosphorylated through the activation of extracellular signal-regulated kinase 1/2 (ERK1/2) signaling by HMGB1 (high mobility group box 1), increases mitochondrial fission, subsequently triggering autophagy activation, which further leads to lysosomal degradation of bone morphogenetic protein receptor 2 (BMPR2) and downregulation of inhibitor of DNA-binding 1 (Id1), ultimately promoting the proliferation and migration of PASMCs (87). The resulting mitochondrial fragments also increase endoplasmic reticulum (ER) stress, further impairing PASMC function (88).

Growing experimental evidence has highlighted the involvement of Mfns, particularly Mfn2, in the development of PAH, largely due to their critical role in regulating mitochondrial fusion. Ryan *et al*. discovered that PGC-1α, an Mfn2 transcriptional coactivator, mediates Mfn2 deficiency in female rats and human PASMCs, causing mitochondrial fragmentation and a proliferation–apoptosis imbalance (89). Researchers have also found a close association between PGC-1α and PPARγ, with the latter's deficiency being viewed as a trigger for insulin resistance, thus linking mitochondrial dynamics dysfunction to metabolic disorders at the molecular level (90). And phosphorylation of Mfn2 is induced by PINK1 (PTEN-induced putative kinase 1) at serine 442, leading to its proteasomal degradation and promoting cell proliferation in PASMC (91). Although Mfn1 and Mfn2 share structural and functional similarities, they differ in their regulatory mechanisms (92). Regulated by miR-125a, Mfn1 is pro-proliferative in hypoxia-induced PASMCs, whereas most Mfns are generally antiproliferative in other vascular beds (14, 92, 93).

In PAH, the balance between mitochondrial fission and fusion is disrupted, resulting in excessive fragmentation. However, we cannot view mitochondrial dynamics solely as an isolated contributor to PAH pathogenesis. Rather, dysregulation of mitochondrial dynamics also affects normal cellular metabolism, maintenance of the cell cycle, and organelle communication. For example, as key regulators of mitochondrial dynamics, Mfns have recently been linked to mitochondrial biogenesis and mitochondrial metabolism (94). Additionally, a 2024 study reported that long-chain acyl-coenzyme A can induce mitochondrial fission, suggesting a potential mechanism for fatty acid-induced fission and expanding our understanding of Drp1 activation (95). Moreover, other regulatory factors, such as microRNAs, play deeply integrated roles in these processes, offering novel therapeutic targets and opening up promising avenues for the future treatment of PAH.

The pathogenesis of PAH involves dynamic interactions between PASMCs, PAECs, and fibroblasts, driven by ER stress and mitochondrial dysfunction. The main functional abnormalities of PAECs include imbalance in the secretion of vasoactive substances, resistance to apoptosis and reorganization of energy metabolism; PASMCs exhibit abnormal proliferation, metabolic reprogramming and phenotypic transformation; fibroblasts participate in the disease process through inflammatory activation and extracellular matrix remodeling. The endoplasmic reticulum-mitochondria interaction plays a crucial role in these cells' pathological changes, including mechanisms such as calcium signal disorder, lipid metabolism abnormality, mitochondrial dynamics imbalance and unfolded protein response.

### 3.2 Dysregulated calcium homeostasis and metabolic reprogramming

MAMs serve as critical hubs for regulating mitochondrial calcium homeostasis (43). VAPB-PTPIP51 tethering proteins regulate autophagy by modulating Ca²^+^ exchange at mitochondria-associated membranes (MAMs), and enhanced ER-mitochondria tethering inhibits mTOR-induced autophagy. This mechanism plays an important role in the proliferation of PASMCs (53, 96). It has been shown that in lung fibroblasts, enhanced interaction of VAPB with PTPIP51 helps to restore the structure of MAMs, thereby reversing endoplasmic reticulum stress and mitochondrial metabolic abnormalities triggered in fibroblast activation (97). And fibroblasts are also considered to be important players in the development of pulmonary hypertension (98). And the dysfunction of SERCA promotes PASMC proliferation by activating the IRE1α/XBP1 pathway in ER stress (99). The same situation occurs in Nogo. The protein modulates the structural organization of the endoplasmic reticulum (ER) and mediates the spatial separation between mitochondria and the ER, thereby regulating inter-organelle communication and functional coordination (32, 100). Its dysregulation can increase this distance, disrupt MAMs, and consequently affect mitochondrial calcium, contributing to metabolic alterations (101). Another mitochondrial protein implicated in calcium transport is UCP2. Its deficiency affects metabolism by inhibiting key calcium-dependent enzymes (102). Additionally, UCP2 deficiency is linked to increased reactive oxygen species generation and reduced NO production in the endothelium, which may be relevant to the pathogenesis of PAH (103). Also, there exists a novel and critical interaction between VDAC2 and eNOS in PAECs, where reduced VDAC2 expression and disruption of the VDAC2-eNOS interaction lead to impaired NO production (104). Changes in intracellular Ca²^+^ homeostasis have also been linked to apoptotic pathways in PAH. For instance, a calcium-activated chloride channel, ANO1, has been identified on the mitochondrial membrane of pulmonary artery endothelial cells (PAECs). Its activation enhances mitochondrial reactive oxygen species (mROS) production, thereby promoting apoptosis (105).

Many researchers compare pulmonary arterial hypertension to cancer, not only because of the cancer-like proliferation of cells and their resistance to apoptosis but also because of the high degree of metabolic similarity between the two (56, 106). We summarize here the main metabolic reprogramming manifestations in PAH and their key enzymes and mechanisms in a table (Table 2). Initially, described to characterize the features of cancer cells, the Warburg effect refers to the shift from mitochondrial oxidative phosphorylation to aerobic glycolysis. This phenomenon has been repeatedly mentioned in research related to pulmonary arterial hypertension (6). Alterations in glucose metabolism in PAH are largely attributed to pyruvate dehydrogenase (PDH) dysfunction. Importantly, mitochondrial Ca²^+^ has been shown to regulate PDH activity (56, 107, 108). This regulatory process involves key proteins such as uncoupling protein 2 (UCP2) and Nogo B (reticulon family member 4B), which modulate mitochondrial Ca²^+^ handling and energy metabolism (101, 109). However, we cannot simply summarize the relationship between calcium and metabolism as a unidirectional mechanism. A study revealed that in the microvascular endothelial cells of PAH patients, elevated ketone levels sensitizes the key calcium signaling channel TRPV4 (transient receptor potential vanilloid 4), thereby disrupting calcium homeostasis (110).

**Table 2:** Main metabolic reprogramming in pulmonary arterial hypertension (PAH).

Metabolic pathway	Key enzymes or proteins	Changes in PAH	Regulatory mechanisms	References
Glycolysis	PDH, PDK, Hk2, PTBP1,	Increased glycolysis to pyruvate and lactate in PAECs and PASMCs	BMPR2 mutations → miR-124↓, PTBP1↑.	(9, 146–148)
			HIF-1α activates PDKs and inhibits PDH.	
Fatty Acid Metabolism	CD36, CPT1, ACACA, BMPR2,	Decreased mitochondrial fatty acid oxidation; Increased fatty acid uptake and storage	BMPR2 mutations → CD36↑.	(149–151)
			CPT1 upregulation in MAMs.	
Glutaminolysis	GLS	Increased glutamine metabolism.	HIF-1α activation.	(152–154)
			SIRT3 regulates.	
Arginine Metabolism	eNOS, ARG1, ARG2	Reduced NO bioavailability; Elevated arginase expression.	Competition between NOS and ARG for arginine.	(155–159)
			HIF-2α-arginase axis.	

The altered metabolic phenotypes observed in PAH are systemic and multifaceted, extending beyond mitochondrial dysfunction and not limited solely to energy supply mechanisms. A critical step in the future development of targeted therapies will be to fully elucidate the interconnections among metabolic pathways, genetic regulation, and substrate utilization.

### 3.3 ER stress

ER stress has emerged as a key player in the pathogenesis of pulmonary arterial hypertension (PAH) (111). Hypoxia and inflammation in PAH can induce ER stress in PASMCs, altering sarcoplasmic reticulum morphology and increasing its separation from mitochondria, ultimately leading to mitochondrial dysfunction (56, 70, 101). Dysregulation of the unfolded protein response (UPR), has been increasingly implicated in PAH progression. Zhuan *et al*. also found that mitochondrial disruption contributes to PASMCs dysfunction by enhancing endoplasmic reticulum stress (88). In addition, ERS-induced inflammation contributes to the development of pulmonary arterial hypertension by promoting pulmonary vascular remodeling, which involves the activation of the PERK/eIF2α/NF-κB signaling pathway (112). IRE1α protein levels were upregulated in hypoxia-induced PASMCs, thereby affecting the role of IRE1α/XBP1 [inositol-requiring enzyme 1(α)/x box binding protein 11] pathway in hypoxia-induced proliferation, migration enhancement, and apoptosis inhibition (113). Activation of ATF6 causes increased levels of Nogo B to further exacerbate endoplasmic reticulum stress-related damage, causing disruption of the mitochondria-endoplasmic reticulum unit (114). HIF-1α, an important factor in the progression of pulmonary arterial hypertension, can exacerbate endoplasmic reticulum stress, while endoplasmic reticulum stress can in turn stabilize HIF-1α, forming a pathogenic feedback loop (56). This interaction severely damages the mitochondria-associated endoplasmic reticulum membrane (MAMs), hinders inter-organelle communication, and accelerates the fragmentation of the mitochondrial network (115). The aforementioned transmembrane proteins have also been utilized as biomarkers for PAH (116). For instance, elevated GRP78 levels have been associated with increased mortality risk in PAH patients, suggesting its potential utility as a prognostic marker (117).

**Figure 2:**
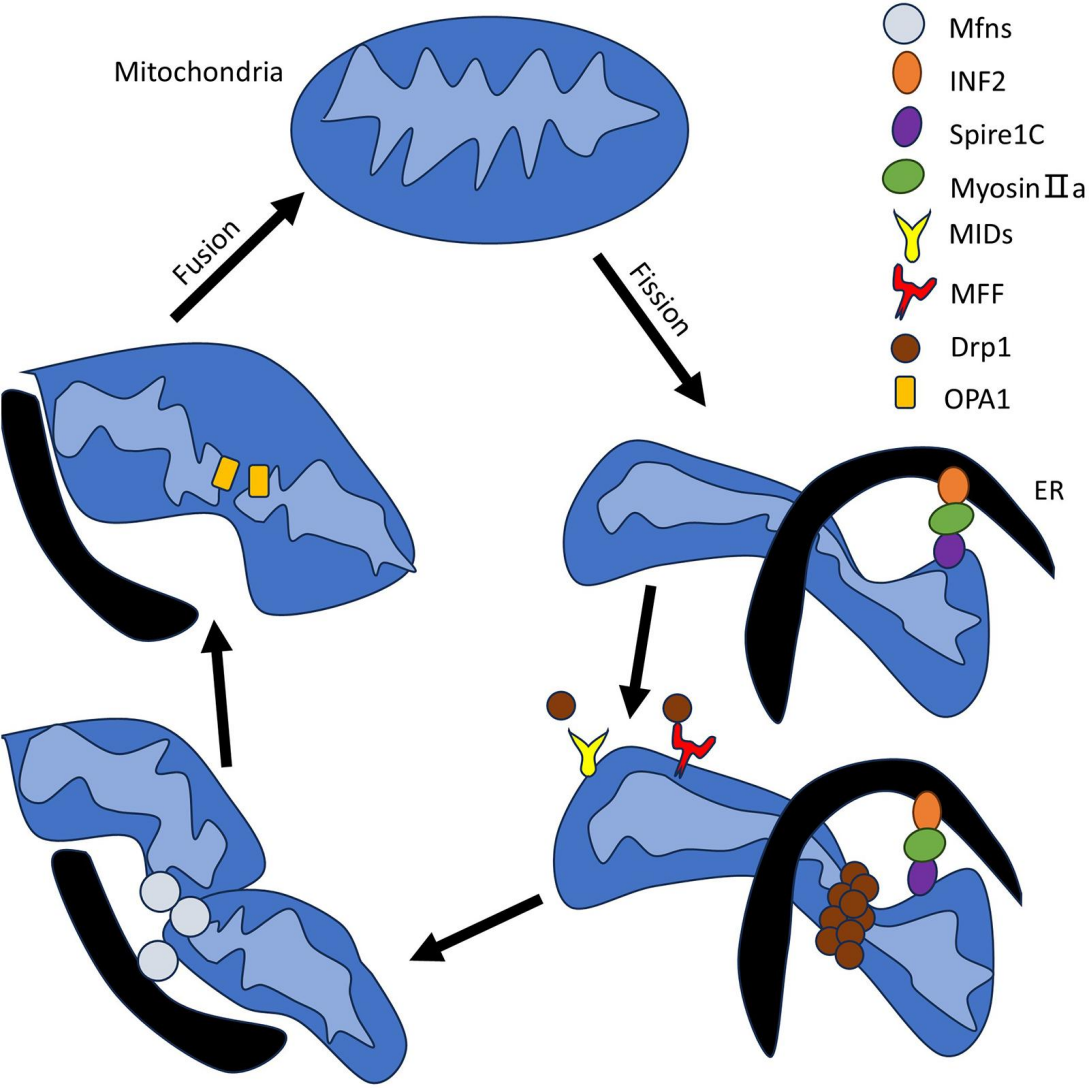
The role of MAMs in PAH in PASMC. This figure is divided into three endoplasmic reticulum modules, each representing a major mechanism: mitochondrial dynamics, calcium homeostasis (metabolism), and endoplasmic reticulum stress (counterclockwise from the upper left corner). Upward arrows indicate an increase, downward arrows indicate a decrease or channel closure, and black arrows connecting different proteins and other substances indicate interactions. The specific mechanisms involved are mentioned in the main text, with corresponding content in Section 3. **Mitochondrial dynamics**: We continue to focus on two key proteins, Mfn2 and Drp1, and detail the specific signaling pathways they participate in within the figure. **Calcium(metabolism)**: The figure lists MAM-localized proteins involved in calcium homeostasis and outlines the functional outcomes of different pathways, including their effects on enzyme activity, apoptosis, autophagy, and endoplasmic reticulum stress. **Endoplasmic reticulum stress**: The figure depicts the three classic pathways of endoplasmic reticulum stress and their downstream effectors. Additionally, we emphasize two key contributing factors—hypoxia and inflammation.

For greater clarity, we used a supplemental figure to express the role of three specific MAMs involved processes in PAH pathogenesis in PASMC, including mitochondrial dynamics, calcium homeostasis, and endoplasmic reticulum stress (Figure 2).

## 4 Therapeutic targets

To provide a comprehensive introduction to mitochondrial-endoplasmic reticulum interactions as potential future therapeutic targets in pulmonary arterial hypertension (PAH), we summarize the currently available pharmacological agents used in PAH treatment in table (Table 3). Over the past few decades, significant progress has been made in developing drugs targeting the three classical dysfunctional signaling pathways in PAH: the prostacyclin, endothelin, and nitric oxide pathways (112). Drugs targeting mitochondrial endoplasmic reticulum interplay in pulmonary arterial hypertension are still mostly studied in cell or animal experiments. However, drug research targeting MAMs has advanced rapidly in other disease contexts, particularly in oncology and neurodegenerative disorders, offering valuable insights into potential molecular targets and therapeutic strategies for PAH (118, 119). Scientists have made progress in regulating mitochondrial function and apoptosis by targeting calcium channel-associated proteins, such as GRP75, IP3R-VDAC1, and MCU (120–122). Other metabolic diseases such as NAFLD (Nonalcoholic fatty liver disease) and PAH have similar dysregulation of calcium homeostasis, disruption of MAMs and endoplasmic reticulum stress (123). For instance, metformin and sulfonamides can improve ER-mitochondrial interactions and structural integrity of MAMs (124, 125).

**Table 3:** Current treatment of PAH.

Category	Drug name	Mechanism/Target
Current	PDE5i	NO-cGMP (160)
	soluble guanylyl cyclase stimulator	
	Endothelin receptor antagonists	Endothelin-1 (161)
	Prostacyclin analogues	Prostacyclin (162)
	Selexipag	PGI2 receptor (163)
	Sotatercept	ACVR2A (164)

A widely used drug for diabetes treatment, the glucagon-like peptide-1 (GLP-1) receptor agonist liraglutide, has been shown to inhibit PDGF-BB-induced proliferation, migration, and dedifferentiation of PASMCs by attenuating pathways such as autophagy, mitochondrial ROS production, and mitochondrial fission. These findings suggest the potential of enhancing mitochondrial and endoplasmic reticulum functional coupling as a therapeutic approach for PAH (126). As mentioned repeatedly previously, pyruvate dehydrogenase, the key enzyme for altered PAH metabolism, was found to reduce mean pulmonary artery pressure and pulmonary vascular resistance, and improve functional capacity after its inhibitor was given to patients with idiopathic PAH (IPAH) in a 4-month study (127). A 2025 study revealed that a Chinese herbal compound, CPG, inhibit pulmonary arterial hypertension (PAH) progression by modulating the Mfn2-IP3R3 signaling axis, which regulates ER stress, mitochondrial Ca²^+^ homeostasis, and autophagy (128). Furthermore, exogenous hydrogen sulfide (H2S) has been shown to reverse PAH by alleviating endoplasmic reticulum stress in both *in vitro* and *in vivo* experimental models (129). Chemical chaperone drugs [e.g., PBA(4-phenylbutyrate) and TUDCA (Tauroursodeoxycholic acid)] are effective in preventing and reversing pulmonary hypertension by inhibiting ATF6-mediated endoplasmic reticulum stress signaling and ameliorating ER-mitochondrial dysfunction and metabolic abnormalities (130). Similarly, the use of fibroblast growth factor (FGF) 21 can alleviate endoplasmic reticulum stress and its impact on endothelial cell apoptosis and dysfunction in hypoxia-induced pulmonary hypertension (131). In addition to these pathways, emerging evidence highlights the complex and often divergent roles of microRNAs and their target gene in the progression of pulmonary arterial hypertension, offering new insights into potentially promising therapeutic strategies (132).

Collectively, accumulating evidence supports the notion that targeting MAMs—particularly through modulation of calcium signaling, metabolic reprogramming, ER stress, and redox balance—holds great promise for the development of novel therapeutics in PAH. As our understanding of MAM structure and function continues to evolve, so too will the potential for designing specific and effective interventions tailored to this critical interface in cellular physiology.

## 5 Future outlook

An improved understanding of the mechanisms regulating MAM integrity or the identification of specific MAMs targets could offer significant therapeutic strategies for PAH. New technologies such as SPLICS (split-GFP-based contact site sensors) allow us to visualize the structure of MAMs more directly and monitor them in real time *in vitro*, potentially opening up new avenues for research (133, 134). It has already shown promise in research on both the SARS-CoV-2 infection (135) and Alzheimer's disease (136). The application of STED (stimulated emission depletion) super resolution microscope in MAM research holds great promise, as its combination with computational modeling enables the observation and reconstruction of more microscopically detailed structures (137, 138). Further development of such tools to study MAM formation in a systemic setting will be invaluable for gaining new insights into the mechanisms controlling MAMs in both health and disease.

## 6 Conclusion

Mitochondria and the endoplasmic reticulum (ER) are intracellular organelles that promote cellular homeostasis by regulating multiple signaling pathways. These interactions influence the pathogenesis of pulmonary arterial hypertension from multiple angles. MAMs (mitochondria-associated ER membranes) serve as bridges between two organelles, are constructed around key proteins, and possess complex functions. Before it can become a new therapeutic target for PAH, further in-depth research into its mechanism is necessary, and this research will continue in the coming years with the development of new technologies.
